# Advanced Ultrasonic Inspection of Thick-Section Composite Structures for In-Field Asset Maintenance

**DOI:** 10.3390/polym15153175

**Published:** 2023-07-26

**Authors:** James A. Quinn, James R. Davidson, Ankur Bajpai, Conchúr M. Ó Brádaigh, Edward D. McCarthy

**Affiliations:** School of Engineering, Institute for Materials and Processes, The University of Edinburgh, Edinburgh EH9 3FB, UK; j.r.davidson@ed.ac.uk (J.R.D.); ankur0062001@gmail.com (A.B.); c.obradaigh@ed.ac.uk (C.M.Ó.B.); ed.mccarthy@ed.ac.uk (E.D.M.)

**Keywords:** non-destructive testing, ultrasonics, delamination, full matrix capture

## Abstract

An investigation into the inspection capabilities of in-field advanced ultrasound detection for use on ultra-thick (20 to 100 mm) glass fibre-reinforced polyester composites is presented. Plates were manufactured using custom moulding techniques, such that delamination flaws were created at calibrated depths. The full matrix capture technique with an on-board total focussing method was used to detect flaws scanned by a 0.5 MHz linear array probe. Flaw through-thickness dimensions were altered to assess the threshold for crack face separation at which delaminations could be identified. Furthermore, part thickness and in-plane flaw dimensions were varied to identify the inspection capability limitations of advanced ultrasonics for thick composites. The results presented in this study demonstrate an inverse relationship between the ability to find delaminations and plate thicknesses, with inspections successful at depths up to 74 mm. When the delamination thickness exhibited surface-to-surface contact, the inspection capability was reduced to 35 mm. There was an exponential decay relationship between the accuracy of the flaw depth measurement and plate thickness, likely due to the necessity of low probe frequencies. The effective inspection depth was determined to be in the range of 1 to 20 times the wavelength. It is speculated that the accuracy of measurements could be improved using probes with novel coupling solutions, and detectors with optimised signal processing/filtration algorithms.

## 1. Introduction

Non-destructive testing (NDT) describes techniques that assess component integrity, without inducing material damage. For asset maintenance, NDT techniques have been incorporated into established operational programmes to evaluate component performance throughout the service’s life period. Current examples include fleet maintenance operations by the Royal National Lifeboat Institution (RNLI) [1,2] and routine ultrasonic testing (UT) of fibre-reinforced polymer (FRP) structures, such as train carriages, minehunters, and submarines, by Babcock International Group PLC [3,4]. The NDT of composite materials is a well-established field, has been comprehensively explored in several literature reviews (for example, in references [5,6,7]), and optimised (for example, in references [8,9,10]). However, the majority of publications focus on thin laminate structures (up to 15 mm thick) commonly found in the aerospace and aeronautical industries. In wind/tidal turbine blades, military vehicles, ships, other sea-going vessels, and structures are often primarily constructed from monolithic FRPs exceeding 20 mm thickness, some of which have been in service for several decades [11,12,13]. A brief summary of research studies relevant to thick-section composite UT is provided as follows.

Ultrasonic testing is a popular NDT technique in which the propagation of ultrasonic waves (typically short pulse waves with centre frequencies in the range of 0.1 to 15 MHz) within a material subject has been observed [14]. For example, features such as cracks [15,16,17,18], delaminations [19,20,21,22], variations in structural and material constitution [23,24,25,26], and manufacturing defects [27,28,29,30] may present as changes in the transmission and reflection energy or changes in the phase of return signals [31]. The term “advanced” when applied to ultrasonics has become broadly accepted in the field to describe a subset of ultrasonic equipment and methods that utilise computerised data collection and processing [32]. Some (non-exhaustive) examples include phased array UT (PAUT), time-of-flight diffraction (TOFD), automated ultrasonic testing (AUT), and total focussing method (TFM) [33,34,35,36,37]. Equipment and methods that fall out from these groupings (such as pulse echo A-scan UT) are occasionally termed “conventional”, for example, see references [38,39,40,41]. Ultrasonic inspection of composite materials is a complex activity, where subject constitution (for example, the fibre volume fraction and/or alignment of anisotropic plies), must be considered. Such variations in the original quality of a composite part will change the material response when subjected to UT, for example, increased porosity will alter the dispersion and bulk velocity properties [42,43]. Furthermore, each component of the composite system will have different acoustic properties (for example, attenuation and wave propagation velocity); therefore, differences in fibre volume fraction between specimens (as global or local parameters, e.g., in the form of resin-rich zones) will bias the global specimen acoustic properties toward those of the more dominant phase [44]. The UT of thick composites presents the particular challenge of requiring the low attenuation and greater penetration ability of smaller inspection frequencies (often ≤1 MHz) due to the usually high damping properties of polymeric materials. However, these low frequencies typically result in reduced spatial resolution [45,46,47].

The NDT of marine composite structures was investigated by Mouritz et al. [48], where a Krautkramer Branson USD15 flaw detector (paired with a Panametrics 0.5 MHz transducer probe) was used to perform the pulse echo A-scan inspection for artificial delamination-style flaws embedded in polyester glass panels. Test specimens ranged from 25 to 150 mm in thickness, and polytetrafluoroethylene (PTFE) film was used to embed flaws of different in-plane dimensions at various depths. Detectable flaws were consistent with damage observed as a result of high-cycle fatigue stresses, such as small (approximately 10 mm) in-plane delaminations, at depths up to 100 mm. Research outcomes from Mouritz et al. provide ideal benchmarks for analysing the performance of the pulse echo A-scan UT with thick FRPs, especially for thicknesses typically utilised in the marine sector. Nevertheless, these are somewhat dated, given the continual development of “advanced” ultrasonic equipment, including detectors, probes, and sophisticated software/analysis tools [49,50].

Subsequently, Battley et al. [51] completed an evaluation of NDT for inspecting marine composites, and considered techniques such as UT, tap testing, and microwave testing. Inspected materials were divided into two categories: real marine structures with pre-existing damage, and manufactured parts with calibrated damage. Sandwich structures with various skin and core thicknesses were predominantly considered, although several monolithic glass FRPs were also evaluated. Instances of the former are listed as follows: glass fibre/epoxy skin with a foam core, glass fibre/epoxy skin with balsa core, carbon fibre/epoxy (pre-preg) skin with honeycomb core, gel coat/glass FRP/plywood skin with balsa core, and glass FRP/Kevlar skin with a foam core. Calibrated delaminations were introduced by embedding PTFE film during laying-up, whilst voids were simulated using heat-sealed polyethylene bags containing dry fibreglass cloth. Both defect types were introduced in four different dimensions and at three unique depths. Notably, UT and microwave testing were able to detect deep flaws in glass FRP up to 16.6 mm thick, whilst tap testing was deemed unsuitable. UT was incompatible with rougher surfaces, which could potentially restrict wider uptakes in marine applications, where course inspection surfaces are common.

A conventional through-thickness UT immersion system was utilised by Balasubramaniam and Whitney [52] in 1996 to characterise the elastic stiffness properties of thick-section glass FRPs. In this study, the descriptor “thick” corresponded to part thicknesses, which were greater than ten times the wavelength of the scanning wave (in this case, up to 28 mm). Utilising pairs of 0.5 and 1.0 MHz transducers, a numerical method was used to find the stiffnesses of inspected composites wherein peak location and time-of-flight data were used to calculate the phase angle and (non-dispersive wave) phase velocity. When compared to conventional methods, measurement errors of the UT technique were observed to be 5–7%. Whilst the examined through-thickness attenuation or immersion techniques have limited applicability in large structures, since both sides of the component may not be accessible, the value of estimating mechanical properties using UT is evident, and the definition of thick composites as a function of wavelength is important for unifying terminology in the proceeding literature.

More recently, Ibrahim published comprehensive reviews of the NDT of thick-section composites [53,54], suggesting that the UT of thick-section composites is immature compared to that of metallic structures, and that NDT techniques are incapable (circa 2016) of full and complete inspections of composite structures. In an article by Taheri and Hassen (2019) [5], the comparative advantages of phased array UT were evaluated for the inspection of glass FRP composites up to 25 mm thick. Finite depth holes of varying diameters were drilled into one side of the panel, and the single element UT (0.5, 1.0, and 1.5 MHz) and array UT (1.5 MHz wedge transducer) were used to inspect from the opposing side. Signal-to-noise ratios were used to evaluate the suitability of each technique, with advanced UT exhibiting 15% increases over conventional UT. As such, the authors concluded that advanced UT detects defects as small as 0.7 mm in diameter; a significant improvement over conventional UT. However, the study by Taheri et al. is restricted to 25 mm thicknesses; further research is required to determine efficacy when structures exceed approximately 25 mm, such as in marine and renewable applications.

A practical assessment of the applicability of various NDT methods for assessing damage in composite structures was compiled by Sheppard et al. [55]. Tap testing, shearography, radiography, microwave testing, thermography, and phased array UT were considered for marine sub-assemblies, consisting of 12 mm thick monolithic glass FRP laminates bonded to structural reinforcement hats. The latter were constructed from non-structural foam with structural polyvinyl chloride cores, and skinned with vacuum-bag cured carbon fibre plies. Phased array UT was performed using a RapidScan 2 system, consisting of a 2 MHz, 64 element, water-filled rubber wheel probe. The resulting A-scans were difficult to interpret, with area coverage being time-consuming due to the small probe contact area. Nonetheless, voids, defects, and inclusions were detectable in the parts, and additional detection in the structural hats on the reverse side was also possible; making it possible to observe any disbonding of these structural hats from the monolithic body. The progress towards advanced ultrasonics with thick FRPs provides an opportunity for detecting flaws with greater accuracy, including the potential for more effective signal filtering to combat the issues of scattering and deflection encountered when scanning composites. Despite these equipment advantages over the research previously discussed, Sheppard et al. only considered a maximum FRP thickness of 12 mm, as is typically found in lifeboats, yachts, and pleasure craft; further research is required to determine efficacy in thicker structures.

Given the current lack of published research, the present study provides a critical analysis of in-field advanced UT of existing thick and ultra-thick monolithic FRPs. The findings contribute toward alleviating premature disposal/decommissioning of large composite components, which is of particular importance given recent concerns regarding sustainability and end-of-life solutions for composite and polymeric materials [56,57,58,59].

## 2. Materials and Methods

### 2.1. Materials

The material system and manufacturing methods used in the present work were selected to represent those of typical marine composite structures. Crystic 489PA isophthalic polyester resin and a cross-linking initiator, Butanox M50 methyl ethyl ketone peroxide (at 2% by volume), were combined and then used to impregnate the reinforcing fibers. The reinforcement used was 800 $g\;m^{-2}$ of plain woven glass mat, supplemented where necessary with 300 $g\;m^{-2}$ of chopped strand glass mat to compensate for the accumulation of crimp and to maintain consistent plate thickness. The curing cycle was 24 h at room temperature (20 °C) with no additional environmental control or post-curing steps.

### 2.2. Manufacturing

**Figure 1:**
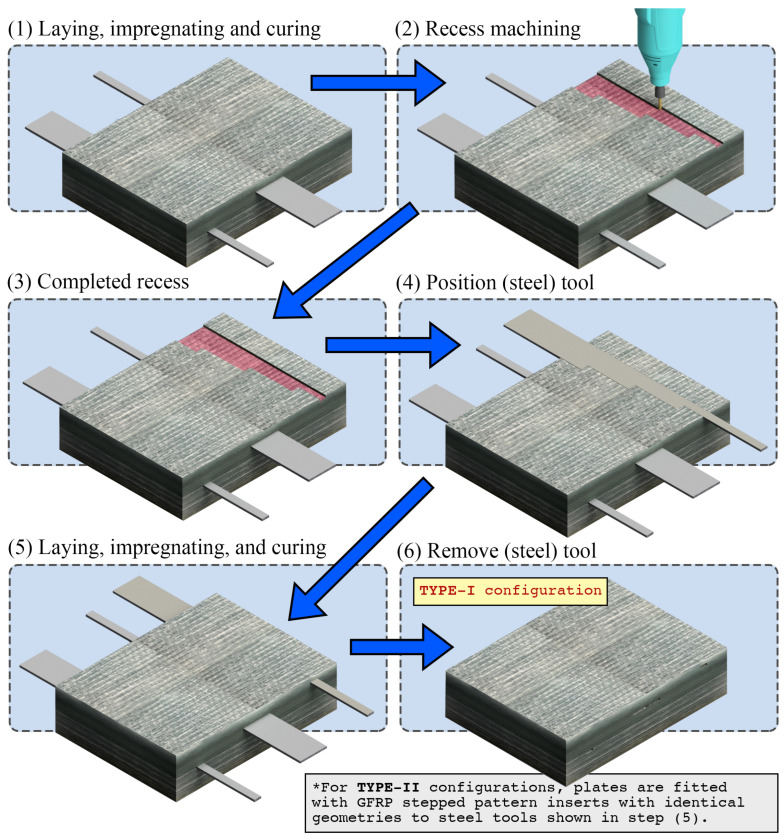
Schematic diagram of manufacturing process.

**Figure 2:**
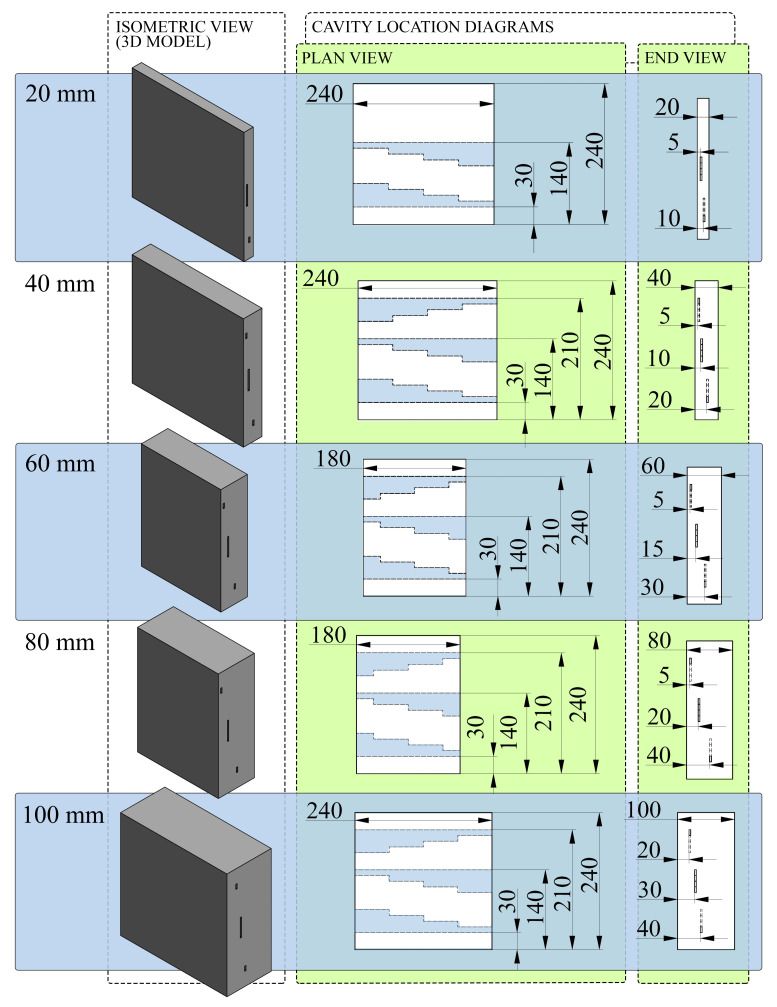
Plate geometry and cavity location diagrams (all dimensions in mm).

Five variations of the glass FRP plate were manufactured, where the panel thickness was increased from 20 to 100 mm, at fixed intervals of 20 mm. The fabrication process consisted of placing fibre mats warp-on-warp, and impregnating the resin mix using a combination of brushes, plastic wedges, and rollers. The fibre volume fraction ($V_{r}$) was controlled in each ply by evenly distributing the liquid resin until a fibre volume fraction of approximately 45% was reached, calculated using Equation (1) (transcribed from ASTM D3171-15), where $M_{r}$ is the mass ratio of reinforcement in the ply, $\rho_{c}$ is the density of the cured composite (1.9 $g\;{\mathrm{cm}}^{-3}$), and $\rho_{r}$ is the density of the reinforcement. Artificial cavities that acted as simulated flaws were created at strategically selected depths, relative to the total thickness—for each panel, as shown in Table 1. The intention of these artificially generated flaw cavities was to simulate in-plane delaminations, which may be developed as a consequence of accumulated in-service damage in real structures. Detecting out-of-plane flaws and/or defects derived from manufacturing remains an equally crucial task; however, the in-plane dimensions of these types of features are often much smaller, resulting in a different set of challenges for successful NDT, compared to the scope of the present work. Cavity locations were selected to generate a full range of (absolute and relative) cavity depths whilst including some relative cavity depths in multiple plates. The process of producing artificial cavities in plates is represented schematically in Figure 1. Cavity formation required the lay-up process to be paused at predefined part thicknesses. After the resin was fully hardened, a series of rotary tools and manual files were used to recess a 3 mm deep stepped shape into the (current) top surface of the part. Steel male counterparts, machined in the same stepwise pattern and coated with Loctite Frekote NC770 mould release agent, were then placed into the recesses. The lay-up was resumed until the next target depth was achieved, or until plate completion. Upon completion of the final curing, the steel tools were removed from the plates, resulting in geometrically consistent cavities. A small draft angle was filed into all sharp edges of the steel tools (e.g., a nylon-headed hammer) could be used to lightly tap the tools out with ease. For all plates, precise geometry diagrams (showing all cavity locations/depths) are given in Figure 2.

(1) $$V_{r}=\left(M_{r}\right)\times 100\times \left(\rho_{c}/\rho_{r}\right)$$

**Figure 3:**
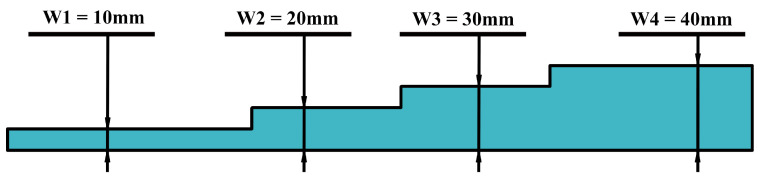
Schematic of the cavity stepwise pattern, including width dimensions.

Three stepped pattern inserts, with identical shapes to that of the steel moulds, were manufactured by the hand lay-up of the same glass FRP system (Figure 3). The in-plane geometry of the glass FRP inserts was machined until a hole-based transition fit (designated 3n14 in ISO 286-1:2010) was achieved—based on sliding inserts within the plate cavities. By utilising glass FRP inserts, the cavity size effect could be explored as an independent variable, with two possible values: no glass FRP insert (3 mm deep cavities), denoted as Type I; and 4-ply glass FRP insert (all-over fixed-transition engineering fit), denoted as Type II. The former acted as a reference case in which UT should be capable of detecting the defects as indicated by existing literature, while the latter simulated delaminations with surface-to-surface contact, resulting from interlaminar shear exhibited after crack formation, without the inclusion of foreign materials such as PTFE.

### 2.3. Testing

#### 2.3.1. Equipment Description

The inspection of calibrated flaws was performed using a Sonatest Veo+ advanced ultrasonic detector paired with a Sonatest X6B-0.5M64E-2x10 (64 elements, 0.5 MHz) linear array probe. The 0.5 MHz probe used was the lowest frequency stock array probe offered by the original equipment manufacturer in the commercial market, and was selected to ensure the greatest possible penetration depth in order to obtain strong Backwall signatures, at the expense of greater resolution. Similar inspection frequencies (≤1 MHz) have been previously used to complete inspections on FRP of similar thicknesses, for example, in references [5,48,52]. Given the comparatively large penetration depth required for this use case (100 mm), relative to typical composite ultrasonic inspections, this compromise was considered favourable. The on-board full matrix capture total focussing method (FMC-TFM) was selected, as this approach completes full-time-of-flight calculations for every focal point and transmitter–receiver combination, exhibiting improved resolution over traditional phased array scanning. A regular cuboidal probe wedge measuring 25×50×130 mm, cast from optical-grade acrylic and coated with a thin film of coupling agent, provided further noise filtration. The coupling agent utilised was a 1:1 (ratio) mixture of the Sonagel Utrasonic Couplant and tap water. A linear encoder calibrated to 16 ticks/mm was used in the scan axis, such that linear sections (denoted as sectors) of the specimens could be displayed in both B-scan and C-scan arrangements.

#### 2.3.2. Data Acquisition

Two discrete plate scanning configurations were considered in this work: (I) plates with no inserts, and (II) plates fitted with glass FRP inserts. The scanning procedure—to be described in the present section—was applied to both cases.

**Figure 4:**
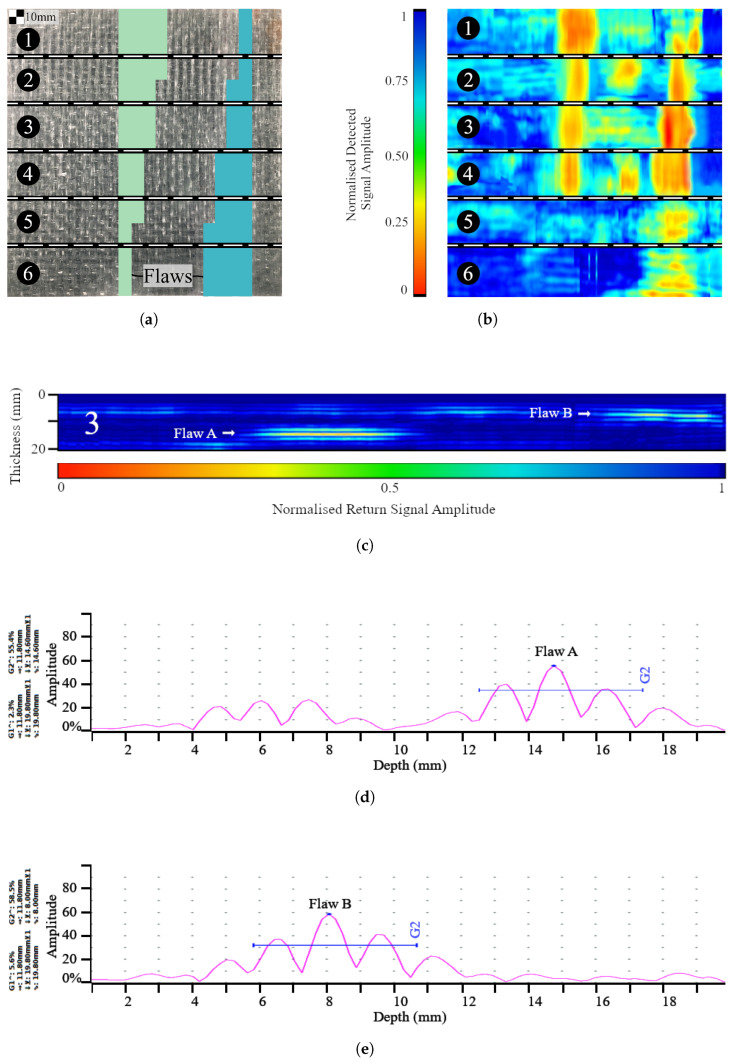
Scanning procedure for representative 20 mm plate: (**a**) Sector locations. (**b**) C-scans. (**c**) Sector 3, B-scan. (**d**) A-scan at Sector 3, flaw A. (**e**) A-scan at Sector 3, flaw B.

Specimens were lightly scrubbed with an acetone towel and placed face up on a clean tabletop. The probe scan width was set to 30 mm, in accordance with manufacturer recommendations based on providing effective focus. Calibration of the detector settings (velocity, gate positioning) was performed using a reference block of the same GRP system, which contained no damage or delaminations, such that the gates were positioned between the front and back wall echos and depth measurements were scaled appropriately. Each specimen was divided into 30 mm wide strips (sectors) on the inspected face using a marker, with each sector numbered sequentially (Figure 4a) to ensure full scan coverage of the specimen. Immediately prior to the initial scanning of each specimen, a calibration procedure was first performed. This consisted of ensuring the detector was programmed with the correct target thickness and appropriate gain values, to maximise feature visibility relative to noise. Due to working memory limitations of the detector in TFM mode, the on-board scan depth was set to half of the part thickness when exceeding 60 mm thick, and affected specimens were scanned twice at each sector (first for the (depth-wise) top half, followed by the (depth-wise) lower half). No further adjustments were applied to the on-board scan settings, on the basis of attempting to replicate real-world use cases where set-up is based on part geometry (thickness, corners, radii, etc.) and the material‘s acoustic properties. This is particularly important since the existence, dimensions, positioning, and depth of flaws/damage are unknown in real-world applications. At the end of each sector, data for A-scans, B-scans, and C-scans were saved and exported for post-processing. This process was repeated for both faces of the plate, thereby doubling the number of depth measurements for each cavity.

#### 2.3.3. Post-Processing

Ultrasound scan data were exported from the detector in the form of native .utdata files, which store the entire dataset (A-, B-, and C-scans) for the given encoded region. These files were post-processed in Sonatest UTStudio+ software, where colour map and software gain were adjusted to output image files of representative A-, B-, and C-scans. The C-scan sectors for each specimen were stitched together using GIMP 2.10.4, effectively creating raster/mapped scans. Determining whether a delamination feature could be identified during the scanning was completed primarily with data from B-scans; the identification of delaminations was noted, both in terms of feature depth and signal amplitude relative to noise in corresponding A-scans. Depth measurements were obtained using gate positioning to ensure consistency across the dataset; in-plane dimensioning was measured as the linear distance travelled by the probe on the plate‘s outer face while the flaw signal amplitude remained above the ambient noise gate.

## 3. Results and Discussion

### 3.1. Representative Scans

**Figure 5:**
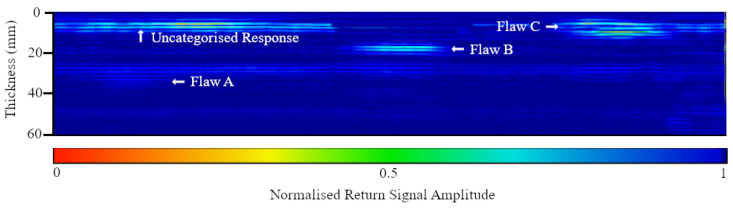
Representative B-scan of the 60 mm specimen.

**Figure 6:**
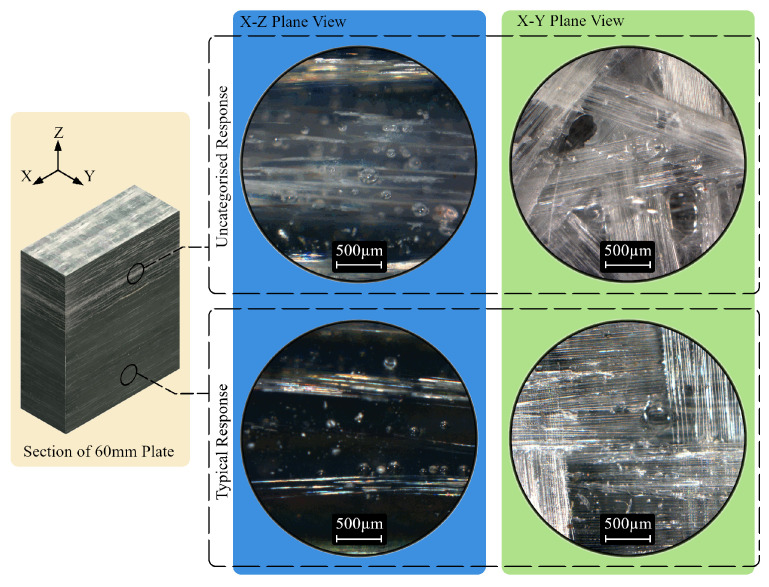
Microscopic examination of scan features in a section cut from the 60 mm specimen.

The first specimen (20 mm thick with Type I flaws) is presented as a case study in Figure 4, showing the scanning methodology (Figure 4a), followed by the corresponding C-scan for each encoded sector (Figure 4b). Sector 3 of that specimen was chosen to display a representative encoded B-scan (Figure 4c), and the corresponding A-scans (when the probe was placed directly above each flaw) are included in Figure 4d–e. Uncategorised variations in acoustic impedance, i.e., experimental noise, were observed in some specimens, characterised by high-amplitude peaks in A-scans and subsequent low signal return regions in B and C-scans, as shown in Figure 5. To verify the status of peaks at these locations as noise rather than delaminations, sections of the affected plates were extracted using a diamond-bladed wet saw, polished, and examined using a Zeiss Axioskop 2 microscope (Figure 6, X-Z plane view). Regions where unexpected variations in impedance were observed corresponded to plies, consisting of short reinforcement fibres, increased void content (for example, air bubbles), and less homogeneous resin dispersion, relative to areas of the plate where typical ultrasound responses were observed. Specimens were manually delaminated at this region to observe the X-Y (in-plane) view of the plies, which were revealed as chopped strand mat plies. By contrast, randomly selected plies were delaminated from the remainder of the specimen and were observed as woven roving mat plies. The amplitude of the waveform from a chopped strand mat region can resemble that of delamination, particularly when the former is closer to the probe than the latter (Figure 5). It may be possible to distinguish between causes of impedance gradients by monitoring signal response waveforms on-board during inspection; however, some modern UT detectors may not have this functionality.

Furthermore, in-field asset inspection is routinely performed on parts of unknown structural conditions; the ability to detect acoustic features without determining causation could lead to misjudgment of an inherent acoustic feature (for example, a resin-rich zone or chopped strand mat region), as a crack, delamination, dis-bond, or other structural damage. It is, therefore, possible for a benign acoustic feature to obscure a damaged region, for example, flaws A and B in Figure 5 are Type I delaminations (3 mm thick) and are easy to overlook during inspection due to the masking effect of the—previously uncategorised—chopped strand mat/experimental noise region.

### 3.2. Plate Thickness

**Figure 7:**
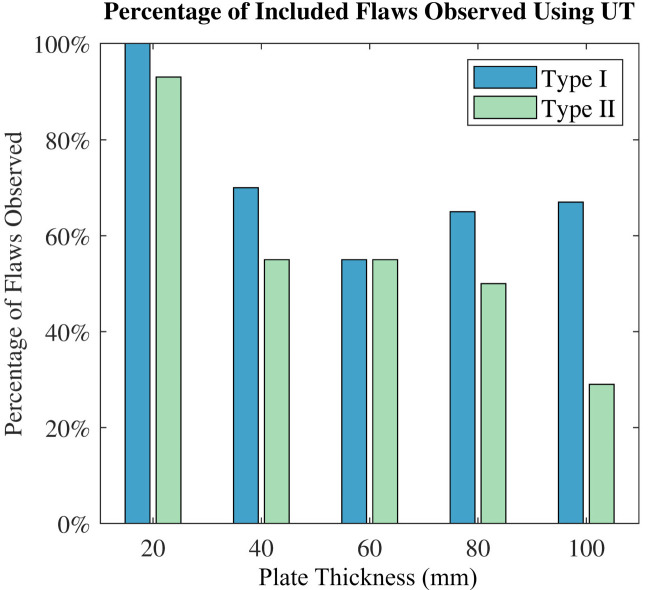
Observability of flaws using UT (Type I and Type II).

The ability to find flaws was assessed by comparing the percentage of flaws found as a function of plate depth (Figure 7). The percentage of flaws observed was herein defined as the ratio of the number of flaws that could be seen to the total number of flaws in a given plate.

For both Type I and Type II flaws, an increase in plate thickness correlates with a general decrease in the percentage of flaws detected; for example, in the 20 mm thick plate, 100% of the Type I flaws and 93% of the Type II flaws were identified. This reduces to 66% and 29%, respectively, for the 100 mm thick plate. The relationship between part thickness and the ability to find flaws was expected since the composite was constructed from two materials that had different acoustic properties (glass and polyester); hence, increasing the ply count through-thickness creates more boundaries where the ultrasound waves refract. The drop in the observation of Type I flaws in the 60 mm thick plate was caused by particularly large peak responses from the CSM regions in that plate, which were often positioned between the detector and the flaw, thereby complicating the task of observing the calibrated flaw peaks; it is surmised that without the presence of these CSM plies, more Type I flaws would have been observed. Furthermore, for the ultrasound signal to penetrate a composite at the thicknesses in the present work necessitates ultra-low frequencies, which reduce sensitivity while increasing attenuation and beam spread [60]. Where flaws are small relative to part thickness (Type II), these factors combine to cause a significant drop-off in detection capabilities, especially as the plate thickness increases. As a direct consequence of the above factors, presently, there is a strong possibility of delamination style flaws in composite laminates greater than 20 mm thick remaining undetectable with present in-field UT technologies, especially where the delaminated crack faces are in contact, independent of the inclusion or positioning of CSM plies.

### 3.3. Flaw Depth

**Figure 8:**
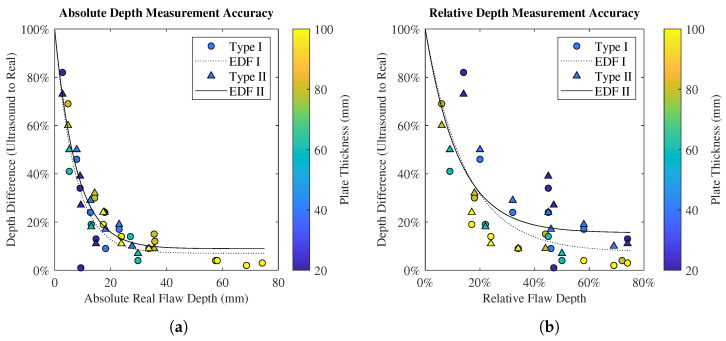
Overall flaw depth measurement: (**a**) Percentage difference between flaw depth measured using UT and real flaw depth as a function of plate thickness, for both Type I and Type II flaws. (**b**) Percentage difference between flaw depth measured using UT and real flaw depth as a function of plate thickness, for both Type I and Type II flaws.

**Figure 9:**
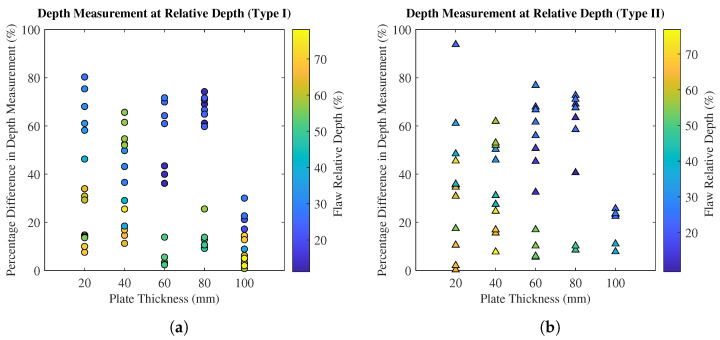
Percentage differences of all observable flaw depth measurements as functions of plate thickness: (**a**) Type I flaws (**b**) Type II flaws.

The present work included a range of real flaw depths, defined as the distance between the external face of the flaw and the probed face (measured with Vernier Calipers). The percentage difference between the flaw depth measured by UT and the real depth is shown in Figure 8a as a function of real, absolute flaw depth. Similarly, the depth difference of the UT measurement as a function of the relative flaw depth is shown in Figure 8b, where the relative flaw depth corresponds to the ratio of the real flaw depth to plate thickness. A least squares optimisation method was used to fit exponential decay function (EDF) trendlines to the data shown in Figure 8; the function is shown in Equation (2), with the parameters A and B listed in Table 2. In order to further probe depth measurement accuracy as a function of plate thickness, the results for all plates are re-plotted in Figure (Figure 9).

(2) $$f\left(x\right)=\left(1-B\right)\exp\left(\frac{-x}{A+B}\right)$$

Figure 8a demonstrates an inverse relationship between real flaw depth and the accuracy of the flaw depth measurement by UT, for both Type I and Type II flaws. The average percentage difference between UT depth measurement and real depth was 22% for Type I and 27% for Type II. The maximum depth at which a flaw could be identified was 74 mm for Type I and 35 mm for Type II. Flaw depth relative to plate thickness is shown in Figure 8b. Irrespective of plate thickness, no flaw can be seen beyond approximately 74% relative thickness, holding for both Type I and Type II flaws.

Some relative flaw depths (such as 45%) were included in several plates, resulting in a range of data points captured at those relative depths. Colour bar scales are used in Figure 9a (Type I) and Figure 9b (Type II) to highlight the depth measurement accuracies as functions of plate thickness where the range of relative depths is clearly displayed. For similar relative depths, further analysis (Figure 9) shows reduced measurement accuracy when scanning less-thick plates (for both Type I and Type II flaws), further reinforcing the inference of an inverse relationship between plate thickness and flaw depth measurement accuracy. The necessity of low-frequency probes to attain signal penetration when inspecting thick composites is well established, and it is speculated that this is—in part—responsible for the reduction in measurement accuracy at shallow flaw depths. In the present work, the inspection was in the range of 1 to 20 times the sound wavelength (the latter being approximately 5 cm); therefore, the waves may not have had the physical distance necessary to develop fully. Furthermore, it is possible that on-board filtration algorithms are better equipped to distinguish between noise and real features when the cavities are at greater depths, owing to a multitude of factors, including noise attenuation and signal-to-noise ratio. When considering less thick plates, no general trend was observed between the type of flaw and measurement accuracy; however, in thicker plates (above 60 mm or 45% relative), the depth measurements of Type I flaws are more accurate than those of Type II flaws. Furthermore, at these plate thicknesses, the percentage of Type II flaws that could be inspected drops significantly compared to Type I (Figure 7).

### 3.4. In-Plane Flaw Dimensioning

**Figure 10:**
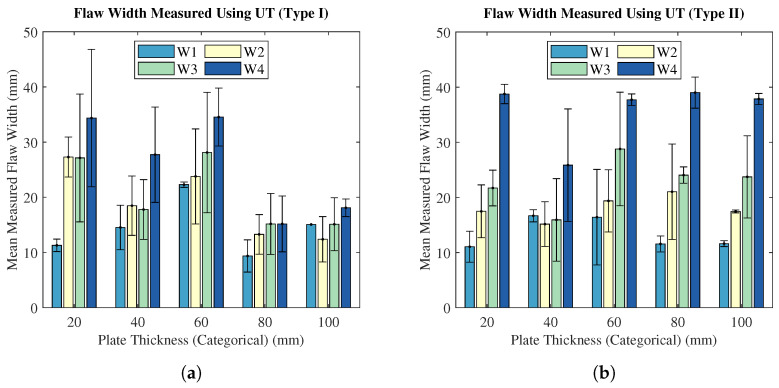
Average UT-measured flaw widths (with standard deviations) at each known flaw width, as a function of plate thickness: (**a**) Type I flaws. (**b**) Type II flaws.

**Figure 11:**
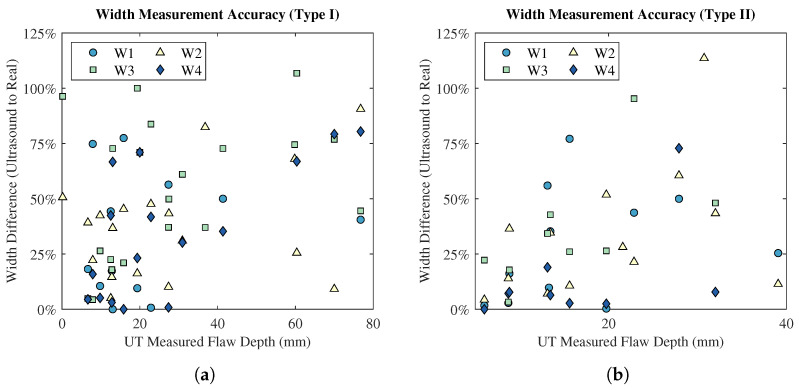
Percentage differences between UT-measured flaw widths and the known, actual flaw widths as functions of UT-measured flaw depth: (**a**) Type I flaws. (**b**) Type II flaws.

The in-plane flaw dimension analysis was completed to evaluate the accuracy of UT as a method for determining the size of a delamination-style flaw in the lamina plane. Figure 10 shows the absolute measured flaw width as a function of plate thickness, whilst Figure 11 displays the accuracy of the width measurement by UT as a function of flaw depth. The accuracy of width dimensioning is defined as the percentage difference between the UT measurement and the known actual flaw width.

Examining Figure 10, no trend exists between the UT-measured flaw width and plate thickness, for both flaw types. The standard deviations of width measurements vary considerably across the test matrix, reinforcing the general inaccuracy of the technique for in-plane dimensioning. Furthermore, the analysis of width measurement accuracy in Figure 11 displays a lack of relationship between in-plane measurement accuracy and flaw depth. Generally, Type II flaws were more accurately measured than Type I flaws; however, there is no statistical significance. Additionally, there were fewer Type II flaws identified than Type I, especially at larger plate thicknesses; therefore, the direct comparison of Type I and Type II is ill-advised in this respect.

## 4. Conclusions

The efficacy of advanced ultrasound for in-field detection of delamination flaws in thick composite sections was assessed using a full matrix capture total focussing method. A variety of delaminations were generated during the manufacturing of glass reinforced polymer blocks, with total specimen thicknesses ranging from 20 to 100 mm, whilst thickness, in-plane dimensions, and depth location were selected as flaw variables. In the present work (i.e., for this material system, specimen construction, and UT system), 3 mm thick flaws were identifiable when embedded at depths up to 74 mm, reducing to 36 mm for surface-to-surface contact delaminations. Regardless of thickness, flaws were observed when embedded at depths up to 74% of plate thickness, beyond which, the signal decay, noise, and mechanically-benign acoustic features limited the success of industrially representative inspection methods. Inverse relationships were observed between specimen thickness and flaw detection, as well as the accuracy of flaw depth measurement and depth of flaw. No trends were observed when evaluating the capability to dimension flaws in-plane, irrespective of delamination size or specimen thickness. Consequently, this advanced ultrasonic inspection system with total focussing methods is effective for detecting delaminations in thick composites (up to 100 mm), provided that the flaw is not deeper than 74% of the part’s depth. However, deeper-set flaws and smaller damage cavities can remain undetected. Furthermore, any feature in the composite that generates a gradient of acoustic impedance with the bulk of the composite (such as a new fibre reinforcement or a resin-rich zone) could be readily misinterpreted as a region of delamination or disbonding.

## Figures and Tables

**Table 1:** Flaw depth locations relative to plate thickness.

Plate	Depth from Front Face			Depth from Rear Face			$V_{r}$ (%)	
Thickness	Flaw 1	Flaw 2	Flaw 3	Flaw 1	Flaw 2	Flaw 3	Mean	SD
20 mm	25%	50%		60%	35%		44.7	8.5
40 mm	12%	25%	50%	80%	68%	43%	44.4	8.3
60 mm	8%	25%	50%	86%	70%	45%	40.4	8.3
80 mm	6%	25%	50%	90%	71%	46%	41.9	11.0
100 mm	10%	20%	30%	77%	67%	57%	46.4	13.8

**Table 2:** EDF trendline parameters for Figure 8.

Figure	Flaw	A	B
Figure 8a	Type I	7.18	0.07
	Type II	7.73	0.09
Figure 8b	Type I	0.14	0.08
	Type II	0.12	0.15

## Data Availability

The data presented in this study are available upon request from the corresponding author.

## Abbreviations

The following abbreviations are used in this manuscript:

NDT	non-destructive testing
RNLI	Royal National Lifeboat Institution
UT	ultrasonic testing
FRP	fibre-reinforced polymer
PAUT	phased array ultrasonic testing
TOFD	time-of-flight diffraction
AUT	automated ultrasonic testing
PTFE	polytetrafluoroethylene
ASTM	ASTM International
FMC	full matrix capture
TFM	total focussing method
CSM	chopped strand mat
EDF	exponential decay function
